# Restrictive type and infectious complications might predict nadir hematological values among individuals with anorexia nervosa during the refeeding period: a retrospective study

**DOI:** 10.1186/s40337-022-00586-x

**Published:** 2022-05-05

**Authors:** Michitaka Funayama, Akihiro Koreki, Yu Mimura, Taketo Takata, Satoyuki Ogino, Shin Kurose, Yusuke Shimizu, Shun Kudo

**Affiliations:** 1 Department of Neuropsychiatry, Ashikaga Red Cross Hospital, 284-1 Yobe, Ashikaga, Tochigi 326-0843 Japan; 2 Department of Neuropsychiatry, Keio University School of Medicine, Shinjuku, Tokyo Japan; 3 Department of Psychiatry, National Hospital Organization Shimofusa Psychiatric Medical Center, Chiba, Japan; 4 Department of Trauma and Critical Care Medicine, Kyorin University School of Medicine, Mitaka, Tokyo Japan

**Keywords:** Anorexia nervosa, Restrictive type, Anemia, Leukopenia, Thrombocytopenia, Pancytopenia, Infection

## Abstract

**Background:**

Although hematological abnormalities in patients with anorexia nervosa have been documented, the mechanisms involved have not been fully clarified, especially during the refeeding period when hematological values further decrease after admission prior to improving. Here we address potential mechanisms underlying the hematological abnormalities of inpatients with anorexia nervosa during the refeeding period.

**Methods:**

We recruited patients from 101 admissions corresponding to 55 individual patients with anorexia nervosa with severe malnutrition (body mass index, 13.4 ± 3.4) from the neuropsychiatry unit in Ashikaga Red Cross Hospital during the period from October 1999 to March 2018. We analyzed three hematological cell measures, i.e., hemoglobin, white cell count, and platelet count, to determine their levels at admission and their lowest levels during the refeeding period and calculated the percent decrease in those values from admission to the nadir levels. We analyzed each measure using a general mixed model with explanatory variables, including data upon admission and a treatment-related indicator, i.e., energy intake.

**Results:**

The initial hemoglobin value of 12.1 ± 2.7 g/dl decreased by 22.3% to 9.4 ± 2.5 g/dl; the initial white cell count was 5387 ± 3474/μl, which decreased by 33.6% to 3576 ± 1440/μl; the initial platelet count of 226 ± 101 × 10^3^/μl decreased by 24.3% to 171 ± 80 × 10^3^/μl. All nadir levels were observed during the refeeding period from the fifth to tenth day of hospitalization. Significant correlations among the three hematological cell measures, particularly at the nadir levels, were found. Of note, 41.7% of our patients who received red blood cell transfusion during hospitalization showed normal hemoglobin levels upon admission. The anorexia nervosa restrictive type was associated with a lower nadir level of white blood cell count. Infectious complications were related to a lower nadir level of hemoglobin and a greater percent decrease in hemoglobin level as well as to the need for red blood cell transfusion.

**Conclusions:**

Nadir hematological cell measures of inpatients with anorexia nervosa might be predicted by the restrictive type and infectious complications. The anorexia nervosa restrictive type was associated with further decrease in hematological values during the refeeding period.

## Plain English summary

Deficiencies in components of the blood, such as a low red blood cell count, low white blood cell count, and low platelet numbers, are observed frequently in patients with anorexia nervosa, particularly those with severe malnutrition, and these deficiencies become manifest after hospitalization during the initial period when patients are reintroduced to food. Why this deterioration occurs even under medical care is not well understood. Here we analyzed the patient factors associated with these blood cell abnormalities. Patients with the restrictive type of anorexia nervosa, and infectious complications were more likely to have the lowest levels of hematological values during the refeeding period.

## Background

Hematological deficiencies, i.e., anemia, leukopenia, and thrombocytopenia, are often found in patients with anorexia nervosa. Their prevalence has been reported to range from 21 to 39% for anemia, 29 to 36% for leukopenia, and 5 to 11% for thrombocytopenia [1]. Recent reports of severely malnourished patients with anorexia nervosa have further provided a higher frequency of hematological abnormalities: 47 to 83% for anemia, 53 to 79% for leukopenia, and 20 to 25% for thrombocytopenia [2–4]. Pancytopenia, a decline in all three hematological measures, is also occasionally found in patients with anorexia nervosa, and its frequency was reported to be 23% among severely malnourished patients with anorexia nervosa [2]. Although anemia itself is considered not to be the immediate cause of death in most cases, anemia is one of the predictors of mortality for patients with anorexia nervosa [5]; the 5-year mortality rate for severely malnourished patients with anorexia nervosa is 11.5%, and their mortality risk is 15 times higher than that in the general population [5].

Hematological deficiencies of patients with anorexia nervosa result from bone marrow hypoplasia characterized by serous atrophy of bone marrow (atrophy of the fatty marrow and loss of hematopoietic cells) that is replaced by an accumulation of gelatinous material (gelatinous marrow transformation) [1, 2]. Bone marrow hypoplasia typically resolve with nutritional rehabilitation [6]. Accordingly, these hematological deficiencies have been associated with malnutrition and its related circumstances in these patients, i.e., a low body mass index [2, 7, 8], the restrictive type of anorexia nervosa [3, 5], and the duration of illness [7]. These previous studies, however, did not control for potential confounding factors.

Most of the previous studies concerning hematological abnormalities in patients with anorexia nervosa used hematological values at outpatient clinics or upon admission. The lowest hematological values, however, are observed during the refeeding period, ~ 1 week after admission [2, 9]. In fact, even though these patients show a V-shaped recovery, their hematological values at the time of discharge are still lower that those upon admission [2]. This is partly because patients with anorexia nervosa are frequently admitted with extracellular fluid volume depletion due to malnutrition and its associated blood plasma decrease or hemoconcentration [2, 7], in which hemoglobin and platelet cell counts per unit of blood volume are elevated [10–12], which makes it difficult to evaluate the hematological deficiencies of these patients upon admission. Furthermore, hematological values might be influenced by infectious complications, in which anemia, leukopenia/leukocytosis, and thrombocytopenia/thrombocytosis were frequently observed [13–18]. In terms of hematological deficiencies, this applies to hemoglobin value because infections complications cause only anemia, but not polycythemia. Infectious diseases are one of the most common complications of anorexia nervosa. According to Guinhut et al. [4], 24.3% of severely malnourished patients with anorexia nervosa developed one or several infectious complications during their hospital stay. This potential influence of infectious diseases on hematological values has not been addressed in previous studies.

Thus, it is crucial for clinicians to precisely predict the nadir hematological levels and the percent decrease in these levels during the refeeding period because patients with anorexia nervosa typically experience nadir values after being refed, suggesting that there might be life-threatening hematological conditions in some cases not only at admission but also during the refeeding period.

However, the factors behind the nadir hematological levels have not been examined. Thus, in this study, we carried out a retrospective investigation of the mechanisms underlying the hematological deficiencies in patients with anorexia nervosa not only at admission but also during the refeeding period, and we evaluated comprehensive variables—including indicators associated with malnutrition, volume depletion, infections complications, and a treatment-associated factor—that potentially influence these hematological values.

## Methods

### Participants

Ethical aspects of this study were reviewed and approved by the Human Research Ethics Committee at Ashikaga Red Cross Hospital. This study was performed after obtaining informed consent from all participants upon admission. For patients below the age of 18 years, informed parental consent was also obtained. Diagnosis was based on criteria in the ICD-10, and each patient was diagnosed by two of the three psychiatrists, each of whom is a board-certified specialist for psychiatry and had > 10 years of experience in psychiatry at the time of the study. Participants were recruited from the neuropsychiatric unit in Ashikaga Red Cross Hospital during the period from October 1999 to March 2018, during which there were 101 admissions with anorexia nervosa (F50.0) from 55 individual patients that were managed in our unit, all of whom had no hematological malignancies. These were categorized into the restrictive type (F50.01, anorexia nervosa, restricting type) and the binge-purge type (F50.02, anorexia nervosa, binge eating/purging type) by two of the three psychiatrists. Among a total of 55 individual patients, all of whom were Japanese, 19 had two or more consecutive admissions, which added up to a total of 101 admissions. These 101 admissions were used for hematological values at admission (Table 1). Regarding the lowest levels and the percent decrease in these three cell measures, we gathered data from admissions for which the lowest level of each blood cell measure was confirmed, that is, individual patients who showed a V-shaped recovery during the refeeding period. Conversely, we excluded the admissions in which each blood cell measure of the individual patients did not show a V-shaped recovery, who were discharged from hospital before reaching their lowest hematological values. We set this exclusion in order to investigate factors contributing to the nadir hematological values. Thus, of 101 admissions from 55 patients, 78 admissions from 45 patients were used for the nadir hemoglobin value, 76 admissions from 43 patients for the nadir white blood cell count, and 75 admissions from 43 patients for the nadir platelet count (Table 1).

**Table 1:** Demographic factors and data at admission and during the refeeding period

Characteristic	Admissions for hematological level at admission (N = 101 from 55 patients)	Admissions for nadir hemoglobin value (n = 78 from 45 patients)	Admissions for nadir white blood cell count (n = 76 from 43 patients)	Admissions for nadir platelet count (n = 75 from 43 patients)
Demographics				
Age (years)	33.4 ± 11.2	35.9 ± 10.4	35.4 ± 10.4	36.1 ± 10.6
Sex (female participants, %)	96.0%	97.4%	97.4%	97.3%
Duration of illness (years)	10.8 ± 9.9	12.1 ± 9.9	12.1 ± 9.8	12.8 ± 9.9
Restrictive type (%)	57.4%	64.1%	63.2%	62.7%
Data at admission				
Weight (kg)	33.8 ± 9.0	31.7 ± 7.7	32.3 ± 7.4	31.4 ± 7.3
Body mass index (kg/m^2^)	13.4 ± 3.4	12.5 ± 2.8	12.3 ± 2.6	12.4 ± 2.6
BUN/Cr ratio	29.1 ± 18.2	30.4 ± 19.4	30.9 ± 19.4	30.5 ± 19.7
Hemoglobin (g/dl)	12.1 ± 2.5	12.1 ± 2.7	N.A	N.A
Mean corpuscular volume (fl)	91.3 ± 9.4	92.1 ± 9.9	N.A	N.A
White blood cells (/μl)	5621 ± 3255	N.A	5387 ± 3474	N.A
Platelets (× 10^3^/μl)	241 ± 106	N.A	N.A	226 ± 101
Caloric intake and weight gain				
Total caloric intake during the first 7 days (kcal/day)	N.A	1183 ± 563	1176 ± 538	1215 ± 563
Weight at discharge (kg)	35.7 ± 7.9	34.1 ± 6.9	34.8 ± 6.7	33.9 ± 6.7
Data during refeeding period (hospital day when nadirs were observed)				
Nadir hemoglobin value (g/dl)	N.A	9.4 ± 2.5 (10.1 ± 9.2)	N.A	N.A
Nadir white blood cell count (/μl)	N.A	N.A	3576 ± 1440 (9.8 ± 10.0)	N.A
Nadir platelet count (× 10^4^/μl)	N.A	N.A	N.A	171 ± 80 (5.5 ± 4.9)

Data represent the mean ± standard deviation

BUN/Cr ratio, blood urea nitrogen / creatinine ratio; N.A., not applicable

### Collection of patient information

Electronic medical records of eligible participants were retrospectively reviewed. As outcome indicators, the following three measures were used: the hemoglobin value, white blood cell count, and platelet count. We determined these values at admission, determined their nadir hematological values during the refeeding period, and determined the percent decrease (the extent of decrease divided by the value at admission). Explanatory variables included demographics (duration of illness, sex, body mass index, presence of chronic kidney diseases, anorexia nervosa subtype, i.e., restrictive or binge-purge), laboratory data at admission (blood urea nitrogen/creatinine [BUN/Cr] ratio and alanine aminotransferase [ALT]), presence of infectious diseases, and an indicator involving treatment, i.e., the amount of caloric intake. Body mass index was calculated as the weight of the individual (in kilograms) divided by the square of the height of the individual (in meters). The BUN/Cr ratio was used to indicate the degree of volume depletion or hemoconcentration. The BUN/creatinine ratio is, however, sometimes inadequate for patients with anorexia nervosa and may be within normal range because malnourished patients may have a low BUN on a nutritional basis [19, 20]. The presence of chronic kidney disease (estimated glomerular filtration rate(GFR) < 60 mL/min/1.73m^2^for three months or longer) was counted as an explanatory variable only for hemoglobin analysis because anemia is a common complication of chronic kidney disease [21]. Values of ALT, an indicator of liver function, were also evaluated as an explanatory variable only for platelet count analysis, low levels of which are often resulted from liver dysfunction [22, 23]. We used values of ALT for the indicator of liver function, but not those of AST (aspartate aminotransferase), because elevated AST values may also be seen in disorders affecting the heart, skeletal muscle, and kidney [24] and patients with anorexia nervosa sometimes present with fatty liver during the feeding period [25], for which ALT is an important biomarker [26]. Because elevation of transaminases in patients with anorexia nervosa is common not only at admission but also during the refeeding period even with a worsening [27–29], ALT values at admission were used for platelet values at admission while the maximum ALT values during the hospital stay were used for the nadir platelet cell counts and the percent decrease in the values. As the ALT values were not normally distributed, they were used for this analysis only after logarithmic transformation [30]. Regarding the presence of infectious diseases, when a patient already contracted an infectious disease at the time of admission, the presence of infectious complications at admission was used as an explanatory variable for hematological values at admission. For hematological values during the refeeding period (the nadir hematological values and the percent decrease in the values), the presence of infectious diseases during the hospital stay (both at admission and during the refeeding period) was counted. As a treatment-related indicator, the caloric intake was measured because it frequently affects laboratory data during the refeeding period [31–33]. Caloric intake (in kilocalories) refers to the average total caloric intake from day 1 through day 7 [31–33], including both oral intake and intravenous infusion therapy. If the patient ate only half the provided 1200-kcal meal, the actual amount of total caloric intake was recorded as 600 kcal. To accurately investigate the effect of energy intake on an individual patient depending on his or her weight, we used the total caloric intake per body weight at admission for this analysis [31, 33]; this measure is widely used for diet therapy for diabetes mellitus [34].

A laboratory panel was carried out upon admission. Regarding tests used for the measurement of the nadir level and the percent decrease in the three blood cell measures as well as the measurement of ALT values during the refeeding period, each blood test from the second examination onward was conducted at 7:30 in the morning before breakfast. The patients frequently underwent serial laboratory tests: 52 admissions (64.2%) were tested on the second hospital day, 43 (53.1%) on the third and fourth hospital days, 35 (43.2%) on the fifth hospital day, 32 (39.5%) on the sixth hospital day, 27 (33.3%) on the seventh hospital day, 31 (38.2%) on the eighth hospital day, 17 (21.0%) on the ninth hospital day, and 14 (17.3%) on the tenth hospital day. The second laboratory panel was carried out for 66 admissions (81.5%) by the fourth hospital day and in 75 admissions (92.6%) by the seventh hospital day. These patients basically continued to have blood tests entered into the data set until their blood cell counts went up or showed a V-shaped recovery. The measurement of vitamin B12, folate, and reticulocytes was not routinely conducted, but were performed as needed.

### Protocol for refeeding

The initial caloric prescription for each patient was determined by the degree of malnutrition, caloric intake preceding admission, and the weight of each patient. Although caloric intake was administered mainly through oral food, intravenous infusion therapy was sometimes used and, less frequently, nasogastric feeding was also carried out. Normally, the total initial caloric prescription consisted of ~ 600–1400 kcal/day and was usually increased by ~ 200 kcal every day. The maximum calorie intake was ~ 3000 kcal/day.

### Statistical analysis

Associations between the three hematological cell measures and patient factors were investigated using values at admission, nadir values during the refeeding period, and the percent decrease within each patient for these values. Regarding data from patients who received red blood cell transfusion, the hemoglobin levels immediately before transfusion were used for their nadir hemoglobin levels. The same applies to data from patients who received platelet transfusion. The general mixed model was used to deal with repetitive admissions, in which individuals were used as random intercepts and other variables as explanatory variables, i.e., demographics (duration of illness, sex, body mass index, and anorexia nervosa subtype), laboratory data (BUN/Cr ratio for all three hematological cell measures and ALT for platelet count), presence of infectious diseases, presence of chronic kidney disease for hemoglobin analysis, and the treatment-related indicator (caloric intake). No single numerical variable had a correlation of > 0.35 with other variables, indicating that all numeric variables were relatively independent, such that all variables were included in the general mixed model. For analysis of the hematological value at admission, the treatment-related indicator was excluded from the explanatory variables. The Bonferroni adjustment was used for multiple comparisons correction by multiplying the *p*-values by the number of comparisons, which was 9 in these analyses. We also investigated associations between the need for red blood cell transfusion and those explanatory variables using mixed effects logistic regression model.

Given the frequent occurrence of pancytopenia, correlations among the three cell measures were also investigated by using multilevel correlation analyses, which were conducted for the values at admission (N = 101), the nadir values during the refeeding period (n = 73), and the percent decrease in the values (n = 73). The latter two cohorts included 73 admissions with confirmed nadir levels for all three hematological cell measures. The Bonferroni adjustment was again used for multiple comparisons correction by multiplying the *p*-values by the number of comparisons, which was also 9. For all statistical tests, two-tailed *p*-values of < 0.05 were considered significant. All statistical analyses were conducted with R (4.1.1).

## Results

Table 1 shows demographic factors and data both at admission and during the refeeding period for admissions with anorexia nervosa. The first column shows data for 101 admissions from 55 individual patients that were used for the hematological values at admission. The other three columns display data for admissions used for the nadir value and the percent decrease of each hematological value: hemoglobin value (n = 78 from 45 patients), white blood cell count (n = 76 from 43 patients), and platelet count (n = 75 from 43 patients). The average body mass index for all admissions was 13.4 ± 3.4, suggesting that this cohort consisted mainly of anorexia nervosa patients with severe malnutrition because a body mass index of < 16 is considered severe malnutrition among patients with anorexia nervosa [35].

The initial hemoglobin value of 12.1 ± 2.7 g/dl decreased by 22.3% to 9.4 ± 2.5 g/dl, which was observed at 10.1 ± 9.2 days after admission; the white cell count at admission was 5387 ± 3474/μl, which decreased by 33.6% to 3576 ± 1440/μl at 9.8 ± 10.0 days after admission; the initial platelet count of 226 ± 101 × 10^3^/μl decreased by 24.3% to 171 ± 80 × 10^3^/μl, which was observed 5.5 ± 4.9 days after admission. The mean corpuscular volume was 91.3 ± 9.4 fl. The number of admissions with mean corpuscular volume < 80 fl (microcytosis) was 7 (6.9%), whereas the number of admissions with mean corpuscular volume > 100 fl (macrocytosis) was 16 (15.8%), suggesting that most admissions (n = 78, 77.2%) involved normocytic normochromic erythrocyte. Among the 16 admissions with macrocytosis, 6 admissions from 5 individual patients were investigated for levels of vitamin B12 and folate, and none had deficiencies for those levels. Of 20 admissions with macrocytic anemia at admission or during the refeeding period, 9 individual patients received assessment of absolute reticulocyte count, the average of which was high at 124 × 10^3^/μl (the normal value: 30–100 × 10^3^/μl [36])), whereas all their hemoglobin values and red blood cell counts were < 10.1 g/dl and < 2.9 × 10^6^/μl, respectively. Among 73 admissions with confirmed nadir levels for all three hematological cell measures, pancytopenia (hemoglobin < 11 g/dl, white blood cells < 4000/μl, and platelets < 100 × 10^3^/μl [37]) was observed in 12 admissions (16.4%). Anemia (hemoglobin < 11 g/dl) was highly prevalent, with 59.4% of admissions having anemia at some point during their hospitalization (60 of 101 admissions). Leukopenia (white blood cells < 4000/μl) occurred in 49.5% of admissions (50 of 101 admissions). Thrombocytopenia (platelets < 100 × 10^3^/μl) was seen in 16.8% of patients (17 of 101 admissions). Although the hemoglobin levels at admission in 4 patients were > 16 g/dl, their hemoglobin levels during the refeeding period decreased to < 13.2 g/dl, suggesting that these patients had elevated hemoglobin levels upon admission, but not polycythemia, for which hemoglobin level for women is > 16 g/dl [38]. Red blood cell transfusion was used to treat 12 admissions (11.9%) from 11 individual patients whose average nadir hemoglobin value was 5.1 g/dl (range: 1.7–7.4 g/dl). Surprisingly enough, the hemoglobin values in 5 cases (41.7%) of these 12 admissions were within normal range (≥ 11.0 g/dl) at the time of admission. Of those 12 admissions, 11 (91.7%) were categorized into the anorexia nervosa restrictive type and 6 (50.0%) had infectious complications. Indeed, the need for red blood cell transfusion was associated with the presence of infectious complications (*p* = 0.02; Odds Ratio = 9.9, 95%CI = 1.5–65.4) along with a lower body mass index (*p* = 0.02). Platelet transfusion was undertaken for two admissions from two individual patients whose nadir platelet cell counts were 4 and 17 × 10^3^/μl, respectively.

Infectious diseases were found in 10 admissions (9.9%) from 9 individual patients (4 admissions from 4 individual patients at the time of admission and 6 admissions from 6 individual patients during the refeeding period, with one patient contracting infectious diseases at different times). Of those 10 admissions, 5 had pneumonia (4 with aspiration pneumonia under bedridden status), 4 had urinary tract infections (2 with catheter-associated urinary tract infections), 2 had central line-associated bloodstream infections, 1 had cholangitis, and 1 had infection with unknown origin, with 3 having two types of infectious diseases at the same time. Disseminated intravascular coagulation [39] was observed in 4 admissions, in which all their platelet counts decreased to < 50 × 10^3^.

Table 2 summarizes the results of analyses with a general mixed model after multiple comparisons correction. The detailed results for each hematological value are displayed in Table 3 for hemoglobin, in Table 4 for white blood cells, and in Table 5 for platelets. The restrictive type of anorexia nervosa was associated with a lower nadir white blood cell count (*p* = 0.001; Table 4) as well as with a lower nadir hemoglobin level (*p* = 0.017; Table 3). A lower nadir white blood cell count was also associated with a longer duration of illness (*p* = 0.019; Table 4). A lower body mass index was associated with a lower white blood cell count at admission (*p* = 0.004; Table 4). The presence of infectious complications was associated with a lower nadir hemoglobin level (*p* < 0.001; Table 3) and a lower nadir platelet count (*p* = 0.024; Table 5) as well as a greater percent decrease in all three cell measures (*p* < 0.001 for hemoglobin [Table 3], *p* = 0.036 for white blood cell [Table 4], and *p* < 0.001 for platelet [Table 5]). A greater percent decrease in the platelet count was also associated with a higher BUN/Cr ratio (*p* = 0.022; Table 5). The presence of chronic kidney disease was associated with a lower nadir hemoglobin level (*p* = 0.009; Table 3). Female sex was associated with a greater percent decrease in the white blood cell count (*p* = 0.043; Table 4). An elevated ALT had a tendency to lower the platelet count at admission (*p* = 0.068; Table 5) and the nadir platelet count (*p* = 0.065; Table 5).

**Table 2:** Factors associated with hematological deficiencies after multiple comparisons correction

Hematological value	Associated with value at admission	Associated with value at nadir count	Associated with percent decrease
Hemoglobin	N.S	Infection*	Infection*
White blood cells	Body mass index**	Restrictive type*	N.S
Platelets	N.S	N.S	Infection*

N.S., the statistical model did not reach statistical significance; * *p* < 0.01; ** *p* < 0.05

**Table 3:** Factors associated with hemoglobin value

Item	At admission			Nadir hemoglobin value			Percent decrease		
	Beta	Standard error	*P*	Beta	Standard error	*P*	Beta	Standard error	*P*
Infection	1.304	1.142	0.257	** − 3.220**	**0.685**	** < 0.001**	**21.94**	**4.800**	** < 0.001**
Chronic kidney disease	− 1.941	1.333	0.149	** − 3.559**	**1.304**	**0.009**	7.218	8.107	0.377
Restrictive type	− 0.827	0.746	0.271	** − 1.787**	**0.725**	**0.017**	4.070	3.504	0.254
Energy intake	N.A	N.A	N.A	− 0.013	0.013	0.319	0.036	0.084	0.672
Female	− 0.242	1.523	0.875	− 1.724	1.786	0.340	14.36	9.461	0.134
BUN/Cr ratio	0.001	0.016	0.944	− 0.012	0.015	0.418	0.114	0.090	0.209
Duration of illness	− 0.023	0.045	0.608	0.011	0.041	0.798	− 0.176	0.195	0.371
Body mass index	− 0.017	0.102	0.866	0.014	0.117	0.900	− 0.924	0.672	0.177

Items with bold formatting are statistically significant factors

N.A., not applicable

**Table 4:** Factors associated with white blood cell count

Item	At admission			Nadir hemoglobin value			Percent decrease		
	Beta	Standard error	*P*	Beta	Standard error	*P*	Beta	Standard error	*P*
Restrictive type	− 578.4	718.6	0.423	** − 1328**	**377.0**	**0.001**	5.941	5.654	0.297
Body mass index	**302.5**	**101.6**	**0.004**	39.61	72.57	0.587	0.346	1.181	0.771
Duration of illness	5.70	45.70	0.901	** − 51.65**	**21.20**	**0.019**	− 0.121	0.324	0.710
Infection	2037	1113	0.071	90.02	440.2	0.839	**17.55**	**8.185**	**0.036**
Female	971.4	1535	0.530	954.0	938.7	0.314	**32.92**	**15.99**	**0.043**
BUN/Cr ratio	22.95	15.74	0.148	9.71	8.965	0.283	0.118	0.156	0.449
Energy intake	N.A	N.A	N.A	0.107	7.483	0.989	− 0.182	0.134	0.179

Items with bold formatting are statistically significant factors. N.A., not applicable

**Table 5:** Factors associated with platelet count

Item	At admission			Nadir hemoglobin value			Percent decrease		
	Beta	Standard error	*P*	Beta	Standard error	*P*	Beta	Standard error	*P*
Infection	− 0.280	4.599	0.952	** − 4.778**	**2.057**	**0.024**	**28.98**	**6.573**	** < 0.001**
BUN/Cr ratio	− 0.036	0.063	0.567	− 0.089	0.045	0.053	**0.316**	**0.135**	**0.022**
Restrictive type	− 3.593	2.725	0.192	− 4.040	2.091	0.059	8.383	5.914	0.164
ALT	− 3.412	1.839	0.068	− 2.604	1.384	0.065	− 5.266	4.335	0.229
Body mass index	0.594	0.390	0.131	0.341	0.355	0.340	− 0.988	1.080	0.364
Female	6.663	5.466	0.230	4.763	5.005	0.347	− 5.665	13.98	0.687
Duration of illness	0.130	0.160	0.422	− 0.044	0.117	0.709	0.364	0.324	0.269
Energy intake	N.A	N.A	N.A	0.022	0.037	0.544	− 0.003	0.116	0.979

Items with bold formatting are statistically significant factors. ALT, aspartate aminotransferase; N.A., not applicable

After multiple comparisons correction, three factors were found to be associated with the hematological values (Table 2). First, the presence of infectious complications was associated with a lower nadir hemoglobin level (*p* < 0.001) and a greater decrease in both the hemoglobin level (*p* < 0.001) and the platelet count *(p* < 0.001). The restrictive type of anorexia nervosa was related to a lower nadir white blood cell count (*p* < 0.01). A lower white blood cell count upon admission was associated with a lower body mass index (*p* = 0.03).

Table 6 shows multilevel correlation coefficient among the three cell measures after multiple comparisons correction. All corrections among the three nadir hematological values were statistically significant (*p* < 0.01). The corrections of the percent decrease between hemoglobin and white blood cell levels as well as between hemoglobin and platelets levels were also significant (*p* < 0.01). Similarly, the correction between white blood cells and platelets levels at admission was statistically significant (*p* < 0.01).

**Table 6:** Multilevel correlation coefficient among the three cell measures after multiple comparisons correction

	Item	Coefficient	*P*
Value at admission	Hemoglobin and white blood cells	0.17	0.756
	Hemoglobin and platelets	0.27	0.099
	White blood cells and platelets	0.43	< 0.01
Nadir value	Hemoglobin and white blood cells	0.43	< 0.01
	Hemoglobin and platelets	0.48	< 0.01
	White blood cells and platelets	0.42	< 0.01
Percent decrease	Hemoglobin and white blood cells	0.39	< 0.01
	Hemoglobin and platelets	0.49	< 0.01
	White blood cells and platelets	0.31	0.063

## Discussion

Three new findings were derived from our present study. First, the lowest hematological values, which were decreased by ~ 25% from those at admission, were associated with the restrictive type of anorexia nervosa and the presence of infectious complications. Second, the presence of infectious complications was also associated with a greater decrease in both the hemoglobin value and the platelet count as well as the need for red blood cell transfusion. Third, significant correlations among the three hematological cell values, particularly at the lowest levels, were also found.

The ~ 25% decrease observed in all three hematological cell measures and their nadir values during the refeeding period are similar to findings of the previous study by Sable et al., in which the average hemoglobin was decreased by 16.0%–10.5 g/dl, white blood cell count by 20.0%–3200/μl, and platelets by 20.1%–214 × 10^3^/μl [2]. In their study, 53 patients with anorexia nervosa who have an average body mass index of 12.9 were recruited [2], which is similar to the average body mass index (13.4) of our cohort. We observed pancytopenia in 17.9% of patients, which is again consistent with their findings that 23% of patients experienced pancytopenia [2], suggesting that deficiencies of all three hematological cell measures are occasionally found in these cohorts. Likewise, significant correlations among the three hematological cell values, particularly at the lowest levels, were also revealed in our present study. These findings suggest that the bone marrow does not function well overall, which is consistent with the presence of bone marrow hypoplasia in patients with anorexia nervosa [1, 2]. Of note, 41.7% of our patients who required red blood cell transfusion during hospitalization showed normal hemoglobin levels upon admission, suggesting a substantial decrease in their values during the refeeding period in some cases. Thus, a re-examination of hemoglobin value after volume restoration is advisable.

The average mean corpuscular volume of 91.3 fl in our cohort was also quite similar to that of the cohort analyzed by Sable et al., in which the mean corpuscular volume was 91.7 fl [2]. According to Sable et al. [2], anemia almost never arose among their cohort due to iron deficiency or vitamin B12 and/or folate deficiency despite extreme dietary restriction and the individuals being underweight for an extended period of time. Furthermore, they postulated that because the vast majority of women with anorexia nervosa have amenorrhea from reversion of their hypothalamic–pituitary–ovarian axis to a prepubertal state, they do not lose blood and iron with monthly menses [2]. In their cohort, 13% of the patients had macrocytic anemia, yet none had vitamin B12 or folate deficiency. We also found that 15.8% of our cohort had macrocytosis at admission and none of the participants had vitamin B12 or folate deficiency, although these laboratory data were not available for all admissions with macrocytosis. Rather, absolute reticulocyte count (i.e., young red blood cells with an ~ 27% larger volume when compared with mature red blood cell [40]) was high when these patients were anemic although these laboratory data again were not available for all admissions with macrocytic anemia. This might suggest that their high mean corpuscular volume levels reflected a larger proportion of reticulocytes, but not macrocytosis due to vitamin B12, folate deficiency, or other causes such as hematological malignancies [41], and that blood formation in bone marrow was accelerated after they were refed, which subsequently led to a V-shaped recovery of hematological values.

The association between malnutrition (body mass index and the restrictive type of anorexia nervosa) and hematological deficiencies in our study is consistent with previous reports [2, 3, 5, 8], although these previous studies did not control for potential cofounding factors. What was not previously known is that this relationship further applies to the hematological values during the refeeding period, in which the lowest hematological cell measures are observed. The restrictive type of anorexia nervosa was found to be a factor that is associated with a lower nadir white blood cell count, suggesting that there might be a distinctive malnutrition pattern in the restrictive type of anorexia nervosa, in which malnutrition is continuous and may be severe when compared with the binge-purge type, even when the body mass index is similar among patients. Indeed, our previous study revealed that the restrictive type independently is associated with the development of refeeding hypophosphatemia, a hallmark of refeeding syndrome, among severely malnourished patients with anorexia nervosa, and this association was independent of a low body mass index and a BUN/creatinine ratio [31]. Given these findings, it is reasonable to consider that the restrictive type itself poses a risk of organ dysfunction and electrolyte depletion.

Infectious complications are closely associated with a lower hemoglobin level, a greater percent decrease in the hemoglobin level and the platelet count, and the need for red blood cell transfusion. The platelet count decline is considered to be resulted from two factors, i.e., the development of disseminated intravascular coagulation [16], in which platelets play an important role in the development of micro thrombosis [42], and the decrease in the platelet count from an elevated level at the peak of an infection to a lower level after recovery [17]. The relationship between infectious diseases and anemia has been proposed in the context of hematopoietic cell regulation [13]. According to Gomes et al. [13], during infection, systemic signals such as inflammatory cytokines can prompt hematopoietic stem progenitor cell (cells responsible for hematopoiesis through self-renewal and differentiation into mature blood cell lineages) to be directed to specific hematopoietic lineages (e.g., myeloid) at the expense of others (e.g., erythroid). Thus, in bone marrow, chronic exposure to inflammatory systemic signals triggers a blockade of erythropoiesis, the process producing red blood cells, and subsequently leads to the development of anemia [13]. For patients with anorexia nervosa whose bone marrow is already hypoplastic, the presence of infectious diseases and its subsequent blockade of erythropoiesis might have a devastating impact on the production of red blood cells. Thus, controlling infectious complications during the refeeding period might prevent further decline in hemoglobin levels. Contracting infectious complications, e.g., aspiration pneumonia under bedridden status, catheter-associated urinary tract infection, and central line-associated bloodstream infection, can be dependent on clinical settings [43–48]. These infections can be preventable or be eased with dysphagia rehabilitation, avoidance of both physical restraint and inappropriate catheter use, appropriate positionings such as the semi-recumbent position, proper aseptic techniques, surveillance, and management strategies [43–49].

### Limitations

Our study has several limitations that should be considered. Fist, although we used the BUN/creatinine ratio for assessment of volume status, this measure is sometimes inadequate for patients with anorexia nervosa and may be within normal range because malnourished patients may have a low BUN on a nutritional basis [19, 20]. Ideally, other measures of volume status, such as the urine specific gravity, should have been employed, which were not available in this study. Thus, the impact of volume status on hematologic values might be more significant than that found in this study. Second, the study population was not large and there is the high possibility of Type 2 error, in particular, the impact of volume status on hematological values. Third, although we tried to investigate the three blood cell measures frequently in each patient, ideally these levels should be examined every day during the refeeding period to precisely determine the nadir levels. Fourth, we were unable to investigate the level of neutrophils and the presence of absolute neutropenia because hematological data of some patients did not include those of neutrophils. In a previous study [2], however, the extent of the decrease in white blood cell count (800/μl) was exactly the same as that of neutrophil count (800/μl) during the refeeding period of patients with anorexia nervosa, suggesting that the change in the white blood cell count might be quite similar to the change in the neutrophil count in patients with anorexia nervosa during the refeeding period. Fifth, this study included data collected over a period of nearly 20 years. Some treatment strategies might have changed, in particular, the adoption of a higher-calorie diet during the refeeding period [50–54]. However, the amount of calorie intake was controlled for in our general mixed model. Finally, the generalizability of our results is limited because our study population was derived from a single hospital. These issues should be addressed and the findings of our study must be confirmed in future large-scale studies.

## Conclusions

Our study found that the lowest hematological values during the refeeding period, which were decreased by ~ 25% from those at admission, were associated with the restrictive type of anorexia nervosa and the presence of infectious complications. The need for red cell transfusion was related to the presence of infectious complications. The anorexia nervosa restrictive type and the presence of infectious complications are considered to cause further decrease in hematological values during the refeeding period.

## Data Availability

The datasets generated and/or analyzed during the current study are available from the corresponding author (MF) upon request.

## Abbreviations

ALT: Alanine aminotransferase
BUN/Cr ratio: Blood urea nitrogen/creatinine ratio
